# Escin Rescues Blood-brain Barrier and Inhibits NLRP3 Inflammasome-mediated Pyroptosis in Rats with Superior Sagital Sinus Thrombosis

**DOI:** 10.7150/ijms.102624

**Published:** 2025-02-28

**Authors:** Min Li, Xinjian Liu, Xinyu Zhou, Ran Meng, Xunming Ji

**Affiliations:** 1 Department of Neurology, Xuanwu Hospital, Capital Medical University, Beijing 100053, China.; 2 Beijing Institute for Brain Disorders, Capital Medical University, Beijing 100069, China.; 3 Department of neurology, The First Affiliated Hospital of Jinan University, Guangzhou 510630, China.; 4 Department of Neurology, Children's Hospital of Soochow University, Suzhou 215025, China.; 5 Department of Neurosurgery, Xuanwu Hospital, Capital Medical University, Beijing 100053, China.

**Keywords:** superior sagittal sinus thrombosis, escin, NLRP3 inflammasome, anti-pyroptosis, blood-brain barrier

## Abstract

**Objective:** The purpose of this study is to investigate the therapeutic value of escin and its potential mechanisms in rats with superior sagittal sinus thrombosis (SSST).

**Methods:** The motor function of rats was assessed using the open field, balance beam, and rotarod tests following one week of escin treatment. Laser Speckle Contrast Imaging (LSCI) was used to evaluate the blood flow in the superior sagittal sinus. Evans blue staining was used to assess the permeability of blood-brain barrier. The protein expression levels of Occludin, ZO-1 and MMP-9 was analyzed by Western blotting to assess blood-brain barrier disruption. Iba1, CD68 and NLRP3 immunofluorescent staining was conducted to evaluate the activation of microglia and NLRP3 inflammasome. The protein expression levels of NLRP3, Caspase-1, IL-1β, IL-18 and GSDMD was analyzed by Western blotting to assess NLRP3 inflammasome activation and pyroptosis. Immunohistochemistry for NeuN was used to evaluate the neuronal pyroptosis.

**Results:** Rats with SSST exhibited decreased movement distance, duration and latency to fall, as well as increased balance latency compared to sham rats, indicating its motor dysfunction. Escin treatment alleviated the motor dysfunction in rats with SSST. LSCI revealed a decrease in the blood flow in the superior sagittal sinus of rats with SSST. There was no discernible change in blood flow between SSST rats treated with escin and those not, suggesting that escin did not promote recanalization of the superior sagittal sinus. By decreasing Evans blue dye content and MMP-9 protein expression and increasing the protein expressions of Occludin and ZO-1, escin treatment improved blood-brain barrier (BBB) disruption and protected tight junction proteins. Escin also reduced the number of microglia-originated macrophage and the number of NLRP3-positive macrophage. Additionally, escin treatment down-regulated the protein expression levels of NLRP3, Caspase-1, IL-1β, IL-18, and GSDMD in the parasagittal cortex of SSST rats, suggesting its ability to inhibit NLRP3 inflammasome activation and therefore reduce pyroptosis. Finally, escin treatment significantly increased the neuronal survival in the parasagittal cortex of rats with SSST.

**Conclusions:** Treatment with escin improved motor function not by recanalizing the SSS. Treatment with escin protected the blood-brain barrier, inhibited the microglia activation and suppressed the NLRP3 inflammasome-mediated pyroptosis in the parasagittal cortex of SSST rats, thereby playing an anti-pyroptosis and neuroprotective effect.

## 1. Introduction

Cerebral venous thrombosis (CVT) is characterized by the presence of thrombosis in the dural venous sinus, cerebral vein, or both, thereby classifying it as a venous stroke^1^, ^2^. CVT accounts for 0.5%-1% of all stroke^3^. The superior sagittal sinus is the most frequently affected site, with 25% to 45% of all CVT cases involving this area^4^. Although the overall prognosis for patients with CVT is favorable, most individuals experienced chronic sequela which impact the quality of life^5^. However, severe CVT is frequently associated with coma, intracranial hemorrhage and venous infarction, resulting in a high incidence of unfavorable long-term outcomes, reaching 56.1%, and a mortality rate of 34.2%^6^. Unfortunately, aside from anticoagulants, there have been limited effective treatment for CVT^7^, ^8^.

Similar to arterial stroke, the inflammatory response also serves as a significant pathological change following CVT^9^, ^10^. In addition, inflammation in parasagittal cortex have the potential to exacerbate the CVT-induced brain damage and contribute to a worse prognosis, making it potential candidates for therapeutic targeting^11^. Pyroptosis is a gasdermin-triggered programmed necrotic cell death which is activated by inflammasome^12^. Excessive pyroptosis causes cytokine storm and detrimental inflammation leading to brain damage. Ding *et al.*^13^, ^14^ and Rashad *et al.*^15^ showed that NOD-like receptor family pyrin domain containing 3 (NLRP3) inflammasome activation may contribute to neuronal injury following CVT and confirmed the occurrence of pyroptosis through animal experimentation.

Escin, a triterpene saponin with ester bonds, is derived from the desiccated and fully developed seeds of Aesculus hippocastanum^16^. Known for its efficacious anti- oedematous, anti-inflammatory and venotonic properties, escin is commonly utilized for the treatment of conditions such as cerebral edema and chronic venous insufficiency, demonstrating a clear therapeutic benefit^17^, ^18^. Nevertheless, the efficacy of escin in treating CVT remains unexplored, with the underlying mechanisms yet to be elucidated.

The utilization of ferric chloride (FeCl_3_)-induced superior sagittal sinus thrombosis (SSST) rat model has been extensively employed in CVT investigations^15^, ^19^, ^20^. The objective of this study is to investigate the potential therapeutic benefits of escin and its effect on the blood-brain barrier and NLRP3 inflammasome-mediated pyroptosis based on Fecl_3_-induced SSST rat model.

## 2. Materials and Methods

### 2.1 Experimental Animals

Two-month-old (200-250 g) male Sprague-Dawley (SD) rats (Beijing Vital River Laboratory Animal Co., Ltd., Beijing, China) were used in this study. Rats were housed in groups of three rats per cage with ad libitum access to water and food and under a 12-h light and 12-h dark cycle. Rats were randomly divided into three groups, namely the sham group, SSST group, SSST + Escin group. Ethical approval was obtained from the Ethics Committee of Capital Medical University, Beijing, China on 10-23-2018 (AEEI-2018-170). Also, this study was performed in accordance with the guidelines of the National Institutes of Health on the care and use of animals.

### 2.2 Modeling of SSST

Rats were weighed, anesthetized by intraperitoneal injection of 2% phenobarbital (50 mg/kg) and fixed in a stereotactic frame (David Kopf Instruments, Tujunga, CA, USA). The calvarial skin was cut along the midline and the skull was exposed. A cranial window of 10 mm in length was prepared along the sagittal suture of the skull with a dental drill to expose the superior sagittal sinus. The drill tip was cooled with normal saline to mitigate thermal injury. The superior sagittal sinus was carefully exposed to ensure that the sinus and dura remained intact. 2-0 silk thread were cut to 10 mm in length, soaked in 20% FeCl_3_ (Cat# I811935, Macklin), and placed over the superior sagittal sinus. After 5min, the silk thread was removed, immersed again in 20% FeCl_3_, and left over the superior sagittal sinus for another 5min. The cranial window was rinsed with normal saline and the skin was sutured. In the sham rats, 2-0 silk thread was soaked in the normal saline.

### 2.3 Treatment with escin

A 3 mg/ml escin solution was made by dissolving lyophilized sodium aescinate powder in normal saline. Rats in the SSST + Escin group received 1 ml of escin solution by gavage twice a day for a week.

### 2.4 Behavioral tests

#### 2.4.1 Open field test

The open field test was conducted to evaluate the spontaneous locomotion. Before each experiment, the rats were acclimated to the equipment room for 30min and the arena was wiped with 75% ethanol. Rats were placed in a 50 x 50 cm closed arena with infrared tracking (Tru Scan Activity System; Coulbourn Instruments, Allentown, PA, USA). Tru Scan 2.0 software (Coulbourn Instruments) was used to analyze the move distance and move time of the rats.

#### 2.4.2 Balance beam test

The balance beam test was used to evaluate the motor balance. The balance beam was placed horizontally 60 cm above the ground, and a 25 × 25 × 25 cm escape box was placed at the end of the balance beam. Rats underwent three sessions of training on the balance beam with an interval of 10 min before testing. During the test, the latency to cross the balance beam, namely the balance latency, was recorded.

#### 2.4.3 Rotarod test

The motor coordination was assessed by the rotarod test. Before testing, rats were trained on the drum of a Rotarod Instrument (Harvard Apparatus, Holliston, MA, USA) for 3 consecutive days to acclimate to speeds of 8, 12, and 16 rpm, respectively. During the test, the Rotarod was set to an accelerating speed from 4 to 40 r/min for 2 min. The latency to fall was recorded and three trials were performed for each rat. The average latency of the three trials was calculated.

### 2.5 Laser Speckle Contrast Imaging (LSCI)

Rats were anesthetized with 2% phenobarbital (50mg/kg i.p.), and the calvarial skin was cut along the midline to expose the superior sagittal sinus. A 1 mm × 1 mm region of interest (ROI) was set on the superior sagittal sinus and the cerebral blood flow in ROI was detected by PeriCam PSI system (v5.0, Perimed, Stockholm, Sweden) with 780 nm laser. The pseudo-color images were obtained by averaging 20 consecutive raw speckle images. Changes in cerebral blood flow are expressed as percentages of the baseline.

### 2.6 Evans blue staining

Evans blue was used as a tracer to assess the permeability of the blood-brain barrier (BBB). 2% Evans blue (Cat# E2129, Sigma-Aldrich) was injected into the marginal ear vein of rats. Two hours later, rats were perfused with normal saline intracardially. The parasagittal cortex was collected, weighted and homogenized. Subsequently, the homogenates were centrifuged at 14,000rpm for 15min and the supernatant was diluted with trichloroacetic acid (1:3). The optical density (OD) at 620 nm was measured with a spectrophotometer (MK3, Thermo Fisher Scientific Inc., Waltham, MA, USA). Evans blue dye concentration was quantified using a standard linear curve and reported as ng of Evans blue dye / g of brain tissue.

### 2.7 Western blotting

Rats were anesthetized (2% pentobarbital, 40 mg/kg i.p.) and sacrificed. The parasagittal cortex was dissected and homogenized in radioimmunoprecipitation (RIPA) lysis buffer (Solarbio, Beijing, China) with protease inhibitors at pH 7.2. The homogenates were centrifuged at 12,000g for 10 min. Protein concentration in the resulting supernatant was measured using the bicinchoninic acid (BCA) protein assay kit (Thermo Fisher Scientific Inc., Waltham, MA, USA). Proteins were electrophoresed in 10% SDS-polyacrylamide gel and transferred to nitrocellulose filter membranes. The membranes were incubated with 5% nonfat milk and the following primary antibodies overnight at 4°C: anti-Occludin antibody (diluted 1: 1,000; Cat# ab216327, rabbit monoclonal, Abcam), anti-zonula occlusionns-1 (ZO-1) antibody (diluted 1:1,000; Cat# SAB5700645, rabbit polyclonal, Sigma-Aldrich), anti-Matrix metalloproteinase-9 (MMP-9) antibody (diluted 1: 1,000; Cat# ab228402, rabbit monoclonal, Abcam), anti-NLRP3 antibody (diluted 1: 1000; Cat# ab263899, rabbit monoclonal, Abcam), anti-Caspase-1 antibody (diluted 1: 1,000; Cat# ab179515, rabbit monoclonal, Abcam), anti-IL-18 antibody (diluted 1: 1,000; Cat# ab191860, rabbit polyclonal, Abcam), anti-IL-1β antibody (diluted 1: 1,000; Cat# ab254360, rabbit polyclonal, Abcam), anti-gasdermin D (GSDMD) antibody (diluted 1:1,000; Cat# 219800, rabbit monoclonal, Abcam), anti-GAPDH antibody (diluted 1:5000; Cat# ab181602, rabbit monoclonal, Abcam). GAPDH was used as internal control. After washing 3 times with tris-buffered saline-Tween 20 (TBST), the membranes were subsequently incubated with the corresponding secondary antibodies for 1 h at room temperature: anti-mouse antibody (diluted 1:10,000, goat monoclonal; Rockland Immunochemicals Inc., Limerick, PA, USA) and anti-rabbit antibody (diluted 1:10,000, goat monoclonal, Rockland Immunochemicals Inc.). The membranes were washed 3 times with TBST. Protein bands were scanned using the Odyssey Infrared Imager (LI-COR Biosciences, Lincoln, NE, USA). A semiquantitative experiment was conducted using the Image J software (Version 1.8.0, NIH).

### 2.8 Immunofluorescence and immunohistochemistry

After anesthesia (2% pentobarbital, 40 mg/kg i.p.) and intracardiac perfusion of normal saline and 4% paraformaldehyde (PFA), brain tissues were prepared and immersed in 4% PFA for 24 h at 4˚C. Brain tissues were embedded with optimal cutting temperature compound. Slices of 30 µm were cut on a cryostat and blocked with 5% nonfat milk at room temperature for 30 min. Slices were incubated at 4 ℃ overnight with anti-Iba1 antibody (diluted 1: 1,000; Cat# 019-19741, rabbit monoclonal, Wako), anti-CD68 antibody (diluted 1: 1,000; Cat# ab955, mouse monoclonal, Abcam), anti-NLRP3 antibody (diluted 1: 1,000; Cat# ab263899, rabbit monoclonal, Abcam) and anti-NeuN antibody(diluted 1: 1,000; Cat# ab177487, rabbit monoclonal, Abcam). Slices were subsequently washed 3 times with PBS. For immunofluorescence, slices were incubated with corresponding Alexa Fluor 488 secondary antibody (diluted 1: 200; Cat# ab150077, goat anti‑rabbit IgG, Abcam; Cat# ab150113, goat anti‑mouse IgG, Abcam) and Alexa Fluor 594 secondary antibody (diluted 1: 200; Cat# ab150080, goat anti‑rabbit IgG, Abcam; Cat# ab150116, goat anti‑mouse IgG, Abcam) at room temperature for 1 h. Finally, slices were counterstained with DAPI (Cat# ab285390, Abcam) and cover-slipped. Images were taken using a Zeiss LSM 800 with Airyscan confocal laser scanning microscope (Carl Zeiss Microscopy GmbH, Jena, Germany) with the same intensity and exposure time at 400X. For immunohistochemistry, slices were incubated with biotinylated secondary antibody (1: 200; Cat# ab64256, goat anti-rabbit IgG, Abcam) for 1 h and at room temperature. Slices were stained using the ABC Kit (Vector laboratories, Burlingame, CA, USA) and the DAB chromogenic kit (Solarbio, Beijing, China). With the slices dehydrated and cover-slipped, images were taken using a KF-PRO-020 digital pathology slide scanner (Ningbo Konfoong Bioinformation Tech, Ningbo, Zhejiang, China). The number of cells were calculated by using Image Pro Plus (Version 6.0, Media Cybernetics, Rockville, MD, USA).

### 2.9 Statistical analysis

The data is shown as mean ± SEM. Since all data are normally distributed, one-way analysis of variance followed by Tukey's post hoc test was employed for group comparisons. SPSS (Version 27.0, IBM Corporation, Armonk, NY, USA) was utilized for statistical analysis and GraphPad Prism (Version 9, GraphPad Software Inc., San Diego, CA, USA) was utilized to create statistical maps. A p value of less than 0.05 was considered statistically significant.

## 3. Results

### 3.1 Escin treatment enhanced the motor function without recanalizing the superior sagittal sinus in rats with SSST

**Figure 1:**
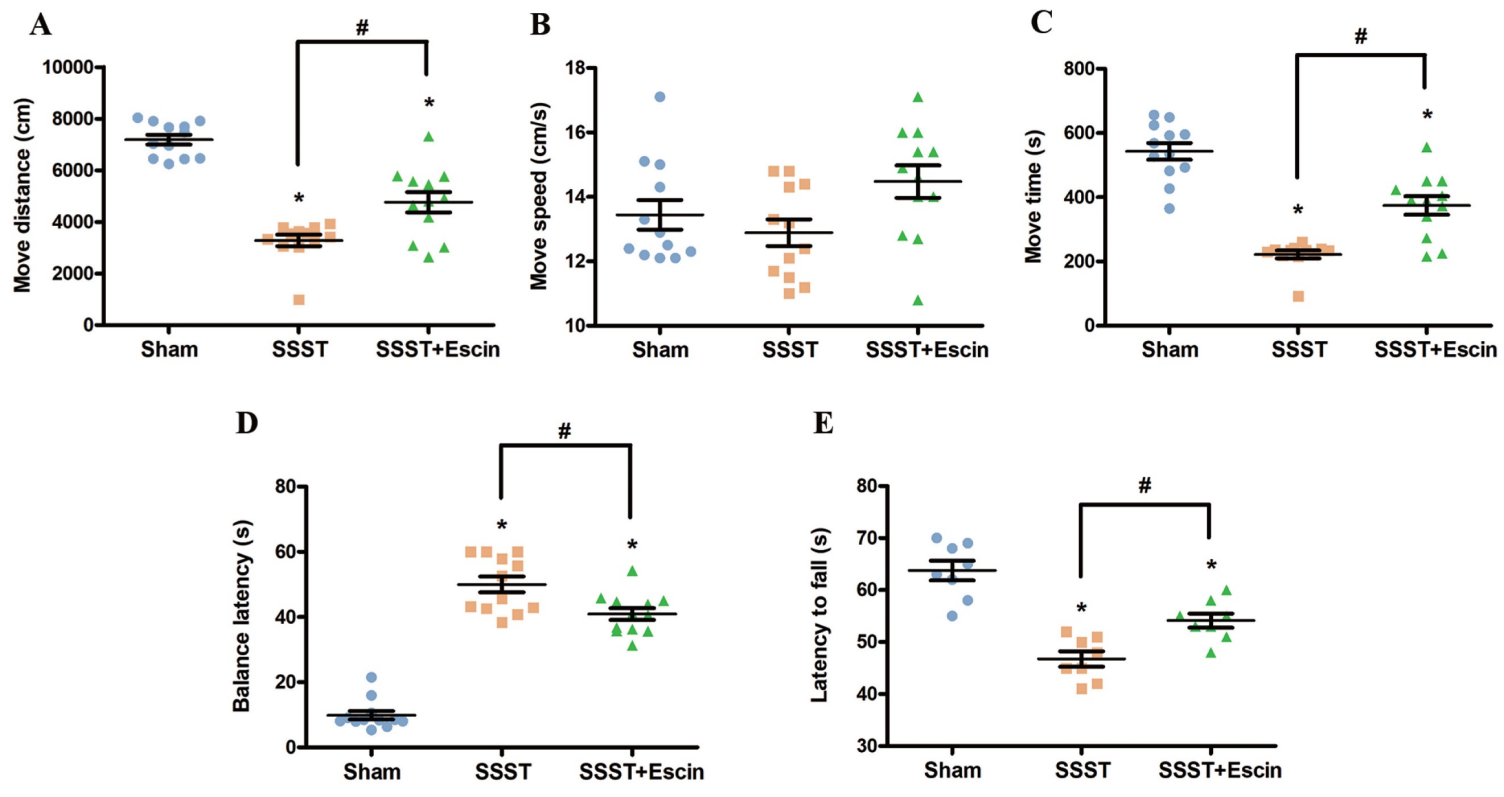
Treatment with escin dramatically increased the movement distance (A) and the move time (C) without improving the move speed (B) in the open field test in rats with SSST. Treatment with escin also reduced the balance latency (D) in the balance beam test and extended the fall-off latency (E) in the rotarod test in rats with SSST. N = 12 in each group. *p < 0.05 *v*s. Sham, # p < 0.05* vs*. SSST.

Behavioral tests were performed to verify the therapeutic effect of escin on motor function of rats with SSST. The SSST group showed decreased move distance and move time in the open field test, reduced fall-off latency in the rotarod test, and prolonged balance latency in the balance beam test compared to the Sham group (Figure 1A-E). Compared with the SSST group, the SSST+Escin group exhibited increased move distance (power = 0.923, effect size = 2.003), move time (power = 0.919, effect size = 1.987) and fall-off latency (power = 0.896, effect size = 1.906), as well as decreased balance latency (power = 0.866, effect size = 3.188) (Figure 1A-E). These findings suggested that escin therapy enhanced the motor function of SSST rats and could be an effective treatment for SSST.

**Figure 2:**
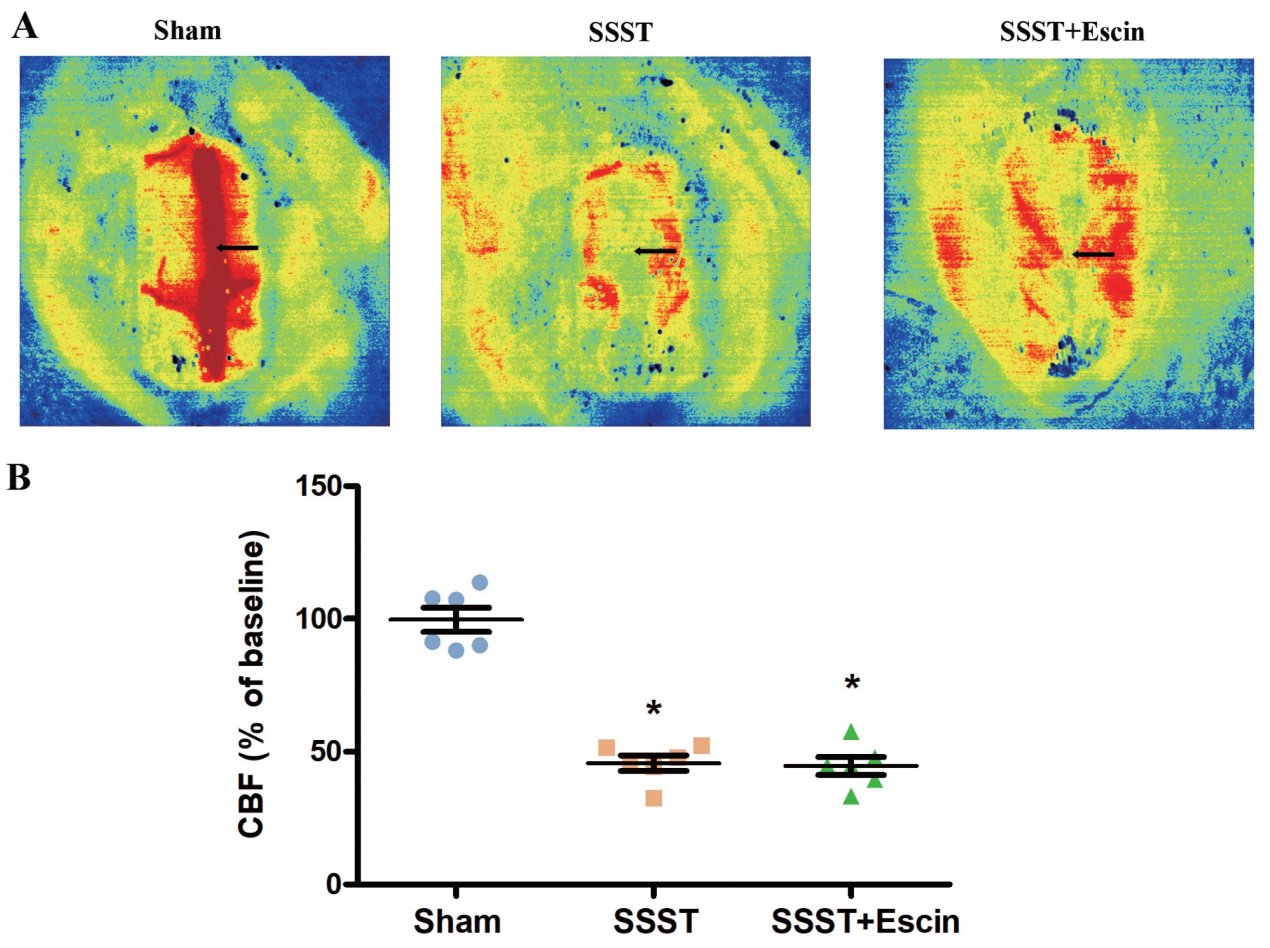
The representative panel (A) and statistical panel (B) of LSCI indicated that escin treatment is unable to remove the thrombus and recanalize the SSS. N = 6 in each group. *p < 0.05 *v*s. Sham.

LSCI assay demonstrated a noticeable decrease in blood flow in the SSST and SSST+Escin groups. No significant difference was found between the SSST+Escin and SSST groups (Figure 2A-B, power = 0.931, effect size = 3.500). These findings indicated a successful establishment of the SSST rat model and suggested that escin treatment failed to remove thrombus or recanalize the superior sagittal sinus.

### 3.2 Escin treatment rescued the BBB in rats with SSST

**Figure 3:**
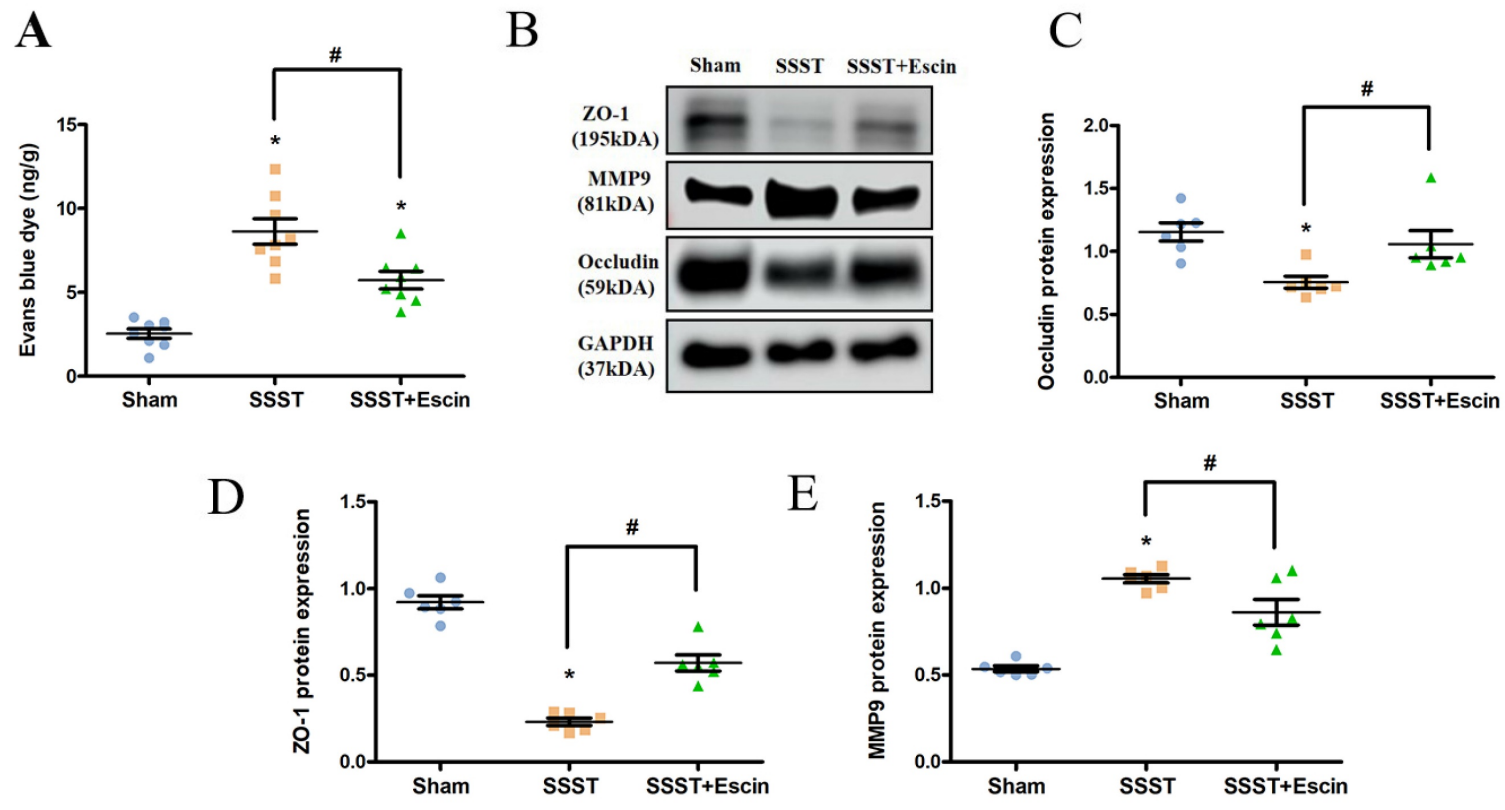
Evans blue staining revealed that escin treatment restored the BBB (A). Western blot analysis showed that escin treatment increased the protein expression levels of Occludin and ZO-1, and decreased the protein expression level of MMP-9 (B-E) in the parasagittal cortex of rats with SSST. N = 8 for Evans blue staining and N = 6 for Western blotting in each group. *p < 0.05 vs. Sham, # p < 0.05 vs. SSST.

Evans blue staining and Western blotting for MMP-9, Occudin and ZO-1 were conducted to evaluate the effect of escin on BBB permeability. The SSST group showed a significant increase in evans blue dye content in the parasagittal cortex compared to the Sham group, whereas escin treatment reversed this increase (Figure 3A, power = 0.906, effect size = 1.940). Furthermore, we found a significant increase in MMP-9 expression and a significant decrease in Occludin and ZO-1 expression in the SSST group compared to the Sham group, indicating damage to the blood-brain barrier in SSST rats (Figure 3B-E). Treatment with escin resulted in a significant decrease in MMP-9 expression (power = 0.978, effect size = 2.349) and a significant increase in Occludin (power = 0.851, effect size = 1.059) and ZO-1 (power = 0.961, effect size = 3.823) expression in SSST rats, suggesting a potential protective effect of escin on the BBB and tight junction (Figure 3B-E).

### 3.3 Escin treatment inhibited NLRP3 inflammasome activation and pyroptosis in rats with SSST

**Figure 4:**
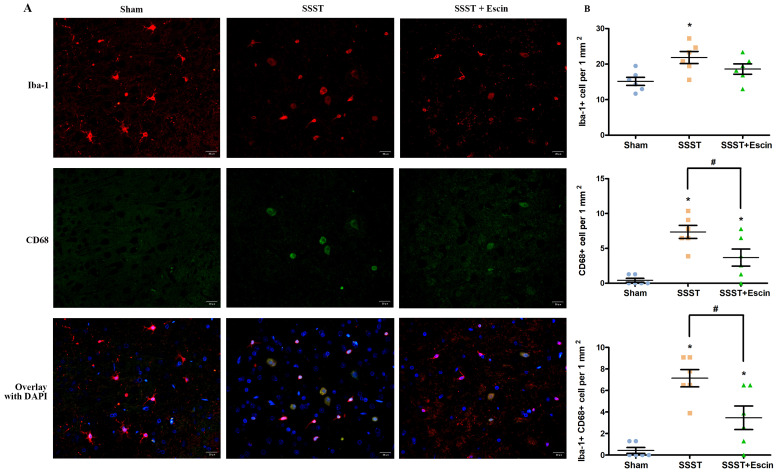
As shown by the representative image (A), treatment with escin significantly decreased the number of Iba-positive (B), CD68-positive (C) as well as Iba-1 and CD68 co-positive cells (D) in the parasagittal cortex of rats with SSST, suggesting that escin treatment inhibited the microglia activation. (A-B) N = 6 in each group. *p < 0.05 *vs*. Sham, # p < 0.05* vs*. SSST.

It is reported that the disruption of BBB may result in the microglial activation and macrophage infiltration, both of which lead to pyroptosis. The SSST group exhibited a higher presence of Iba1-positive, CD68-positive as well as Iba1 and CD68 co-positive cells in the parasagittal cortex compared with the Sham group, indicating an increase in microglial activation (Figure 4A-D). Administration of escin resulted in a reduction of Iba1-positive cells (power = 0.838, effect size = 0.948), CD68-positive cells (power = 0.922, effect size = 1.567) as well as Iba1 and CD68 co-positive cells (power = 0.824, effect size = 1.714), suggesting that escin exerted an inhibitory effect on microglia activation. (Figure 4A-D).

**Figure 5:**
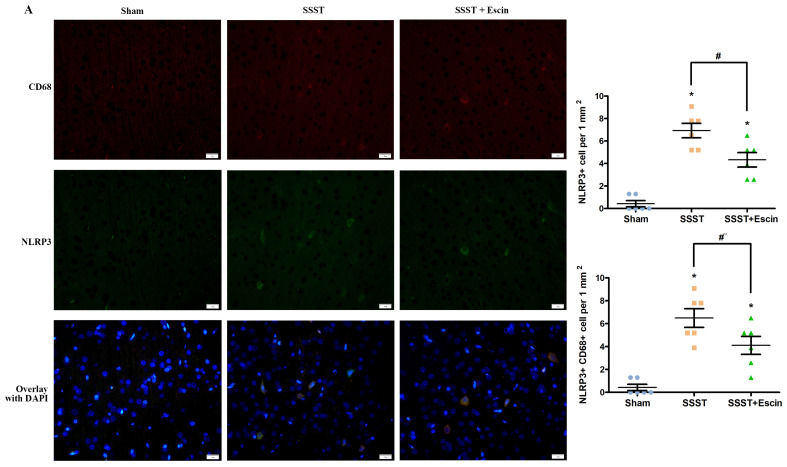
As shown by the representative image (A), treatment with escin significantly decreased the number of NLRP3-positive cells (B) as well as NLRP3 and CD68 co-positive cells (C) in the parasagittal cortex of rats with SSST, suggesting that escin treatment inhibited the activation of NLRP3 inflammasome in activated microglia. (A-B). N = 6 in each group. *p < 0.05 *vs*. Sham, # p < 0.05* vs*. SSST.

In addition, the SSST group exhibited an increased number of NLRP3-positive as well as CD68 and NLRP3 co-positive cells in the parasagittal cortex compared with the Sham rats, indicating a higher level of NLRP3 inflammasome activation in activated microglia (Figure 5A-C). Escin treatment reduced the number of NLRP3-positive cells (power = 0.985, effect size = 2.437) as well as CD68 and NLRP3 co-positive cells (power = 0.878, effect size = 1.852), suggesting that escin exerted an inhibitory effect on the activation of NLRP3 inflammasome in activated microglia (Figure 5A-C).

**Figure 6:**
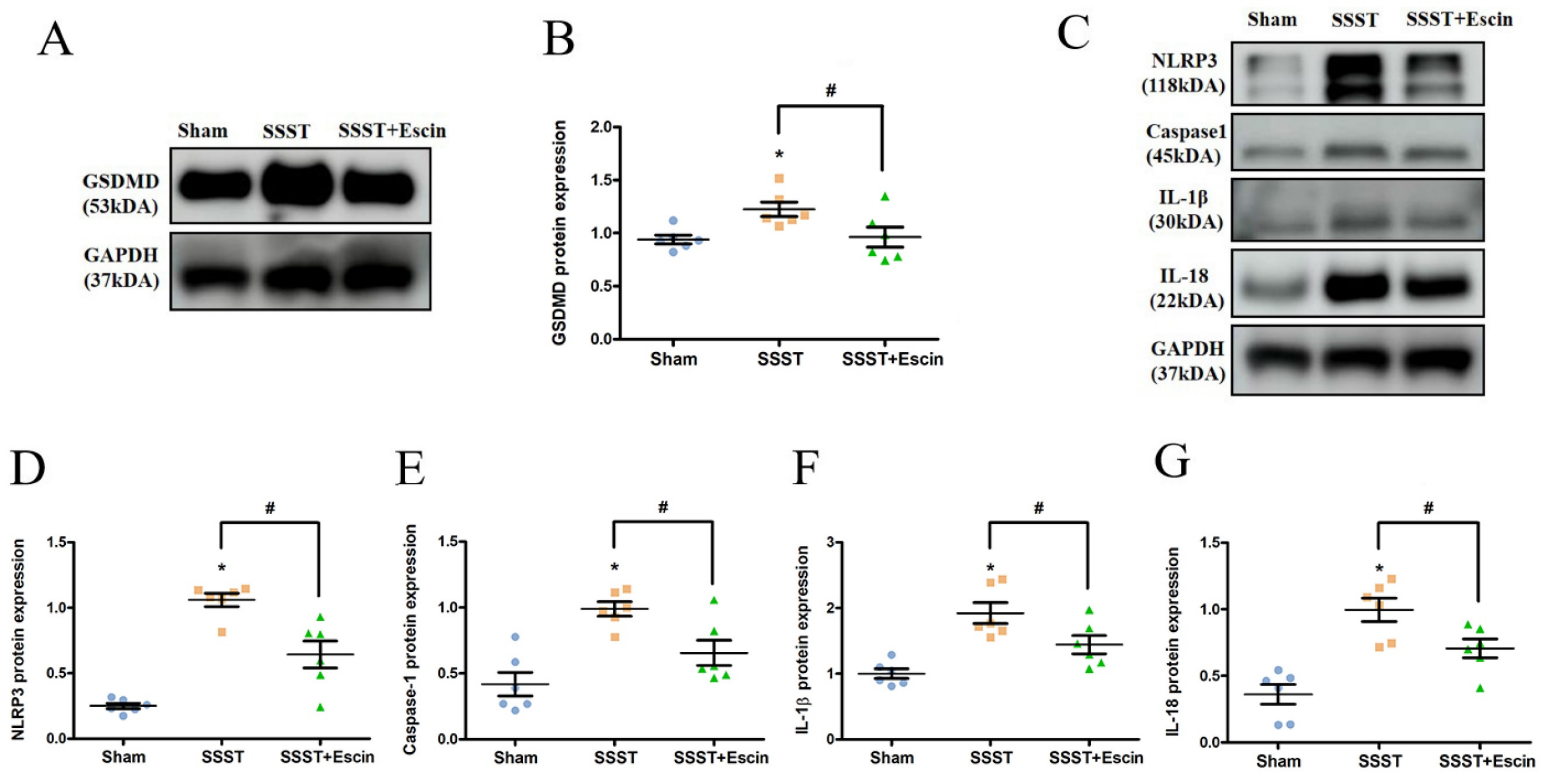
Results from the Western blotting showed that escin treatment significantly decreased the protein expression levels of GSDMD (A-B), NLRP3, Caspase-1, IL-1β and IL-18 (C-G) in the parasagittal cortex of rats with SSST. N = 6 in each group. *p < 0.05 vs. Sham, # p < 0.05 vs. SSST.

In order to explore the impact of escin treatment on the NLRP3 inflammasome pathway, the protein expression levels of GSDMD, NLRP3, Caspase-1, IL-1β and IL-18 were analyzed. The SSST group exhibited elevated protein levels of GSDMD, NLRP3, Caspase-1, IL-1β and IL-18 compared to the Sham group, indicating activation of the NLRP3 inflammasome in the parasagittal cortex of SSST rats and promotion of pyroptosis (Figure 6A-G). Escin treatment reduced the protein expression levels of GSDMD (power = 0.808, effect size = 0.912), NLRP3 (power = 0.986, effect size = 2.454), Caspase-1 (power = 0.865, effect size = 1.424), IL-1β (power = 0.882, effect size = 1.463) and IL-18 (power = 0.802, effect size = 1.667), suggesting that escin inhibited NLRP3 inflammasome activation and depressed pyroptosis (Figure 6A-G).

**Figure 7:**
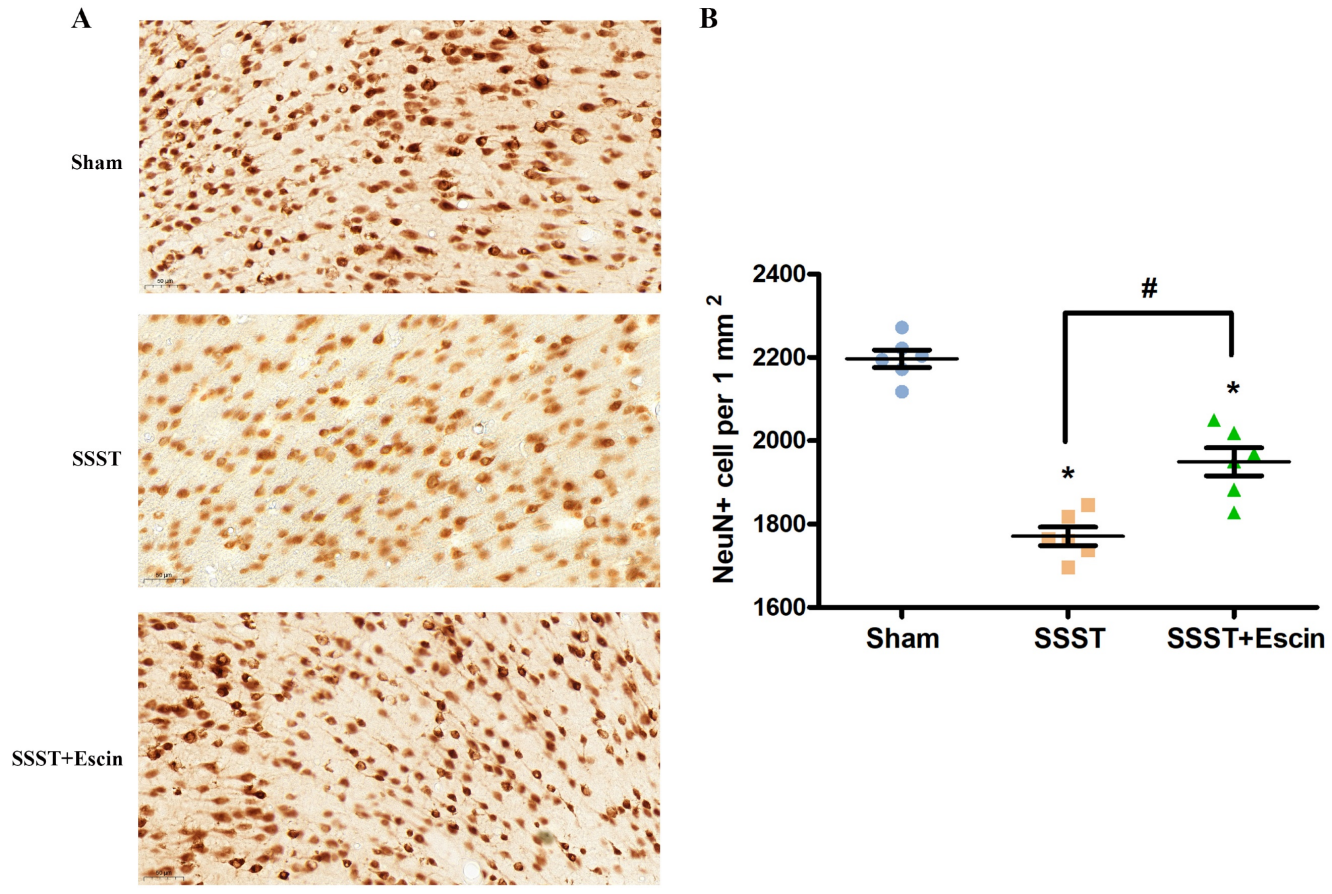
As shown by the representative image (A), treatment with escin significantly increased the number of surviving neurons in the parasagittal cortex of SSST rats (B). N = 6 in each group. *p < 0.05 *vs*. sham, # p < 0.05.

The NeuN immunohistochemistry was conducted to investigate the neuroprotective effect of escin. The number of surviving neurons in the parasagittal cortex was significantly reduced in the SSST group compared with the Sham group (Figure 7A-B). Escin treatment significantly increased the number of surviving neurons in rats with SSST, confirming the neuroprotective effect of escin (Figure 7A-B, power = 0.907, effect size = 3.322).

## 4. Discussion

The results from behavioral tests showed that escin treatment significantly improved the motor function of SSST rats, indicating a neuroprotective role of escin treatment on CVT. The results from LSCI indicated that escin treatment is unable to remove the thrombus and recanalize the superior sagittal sinus. We demonstrated that the neuroprotective role of escin was not associated with the recanalization of the superior sagittal sinus. Although there is a trend, several studies 19, 21 have confirmed that the neurological performance of SSST rats was statistically independent from the degree of recanalization. It is suggested that factors other than recanalization may potentially have an impact on the recovery from neurological deficit. Our results are the first to indicate that escin inhibited the NLRP3/Caspase-1/GSDMD-mediated pyroptosis by protecting the BBB, inhibiting microglial activation and reducing infiltration of macrophages.

Disruption of the BBB is a significant pathophysiology of CVT^15^, ^22^. Results from Evans blue staining confirmed the disruption of BBB following CVT. In addition, we demonstrated that escin treatment restored the BBB. The BBB is maintained by the tight junctions which are composed of cytoplasmic accessory protein such as ZO-1 and integral membrane protein such as Occludin^23^. The decrease of tight junction proteins is closely related to the BBB disruption and the recovery of tight junction proteins is associated with restoration of BBB^24^. In this study, escin treatment up-regulated the protein expression of Occludin and ZO-1 in rats with SSST. Over-production of the matrix metalloproteinase, particularly MMP-9, lead to degradation of collagen and laminin in vascular basement membranes. Inhibition of MMP-9 contributes to the restoration of BBB^25^. A study by Sun *et al.*^26^ reported that escin protected the BBB by reducing MMP-9 production through activation of the AMPK-Cav-1 pathway. Results of this study also revealed that escin treatment inhibited the MMP-9 production in rats with SSST. Therefore, it is suggested that escin protected the BBB by inhibiting MMP9 production.

It is well-accepted that neutrophils, monocytes and macrophages aggregate in the venous wall following CVT and infiltrate into the brain parenchyma after BBB breakdown^11^, ^27^. The release of pro-inflammatory factors then triggers microglial activation and exacerbate inflammatory response^11^. A study by Hu *et al.*^28^ have shown that escin inhibited neutrophil adherence and activation. The cells that were co-positive for microglia marker Iba-1 and macrophage marker CD68 were activated microglia with macrophage activity. In this study, we observed an increased number of Iba-1 and CD68 co-positive cells in the parasagittal cortex of rats with SSST, indicating an enhanced microglial activation. We also discovered that escin treatment significantly inhibited the microglial activation.

Pyroptosis is an inflammatory programmed cell death. NLRP3 inflammasome in inflammatory cells plays a crucial role in the pathogenesis of pyroptosis^29^. NLRP3 inflammasome triggers pro-Caspase-1 to form Caspase-1. Caspase-1 subsequently cleaves pro-interleukin-1 β (pro-IL-1β) and pro-IL-18 into bioactive IL-1β and IL-18. Caspase-1 also cleave GSDMD into N-terminal GSDMD (GSDMD-N) and C-terminal GSDMD (GSDMD-C). GSDMD-N binds to the plasma membrane to form membrane pores which facilitate the release of IL-1β and IL-18. The latter two compounds exacerbate the inflammatory cascade^30^. Activated microglia have been shown to display both GSDMD and NLRP3 immunoreactivity in earlier investigations^14^, ^15^. Elevated protein levels of NLRP3, Caspase-1, IL-1β and GSDMD was also observed in the cortex of the SSST rat model and the SSST mice model^13^, ^14^. Results from this study confirmed an increased number of NLRP3-postive activated microglia as well as an increased protein expression levels of NLRP3, Caspase-1, IL-1β, IL-18 and GSDMD in the parasagittal cortex of rats with SSST. Remarkably, escin treatment reversed the elevated NLRP3, Caspase-1, IL-1β, IL-18 and GSDMD. Furthermore, results from NeuN immunohistochemistry showed that escin treatment increased the neuronal survival. It is suggested that escin exhibited anti-pyroptosis effect and neuroprotective effect by inhibiting NLRP3 inflammasome activation in the parasagittal cortex of SSST rats.

The findings of this study elucidated the translational potential of escin in clinical practice. If future clinical trials support the findings of this study, CVT patients may benefit from escin add-on treatment. However, there were limitations of this study. First, we only demonstrated that escin has an anti-inflammatory impact via inhibiting the NLRP3 and MMP-9 inflammasomes, but did not find out the exact molecular pathway through which escin acts on the MMP-9 and NLRP3 inflammasome. Second, we did not observe the long-term therapeutic effect of escin on rats with SSST. Future research evaluating the long-term therapeutic effect of escin will be carried out. Third, although the FeCl3-induced SSST rat model is commonly used, it does not fully replicate human disease progression. Since escin is a post-marketing medication, we will carry out a randomized controlled trial in the future to confirm the therapeutic effect of escin in CVT patients.

## 5. Conclusions

Treatment with escin improved motor function not by recanalizing the SSS. Treatment with escin protected the blood-brain barrier, inhibited the microglia activation and suppressed the NLRP3 inflammasome-mediated pyroptosis in the parasagittal cortex of SSST rats, thereby playing an anti-pyroptosis and neuroprotective effect.
